# Gingerol-Enriched Ginger Supplementation Mitigates Neuropathic Pain *via* Mitigating Intestinal Permeability and Neuroinflammation: Gut-Brain Connection

**DOI:** 10.3389/fphar.2022.912609

**Published:** 2022-07-08

**Authors:** Chwan-Li Shen, Rui Wang, Vadim Yakhnitsa, Julianna Maria Santos, Carina Watson, Takaki Kiritoshi, Guangchen Ji, Abdul Naji Hamood, Volker Neugebauer

**Affiliations:** ^1^ Department of Pathology, Lubbock, TX, United States; ^2^ Center of Excellence for Integrative Health, Lubbock, TX, United States; ^3^ Center of Excellence for Translational Neuroscience and Therapeutics, Lubbock, TX, United States; ^4^ Department of Pharmacology and Neuroscience, Lubbock, TX, United States; ^5^ Department of Medical Education, Lubbock, TX, United States; ^6^ Department of Microbiology and Infectious Disease, Lubbock, TX, United States; ^7^ Garrison Institute on Aging, Texas Tech University Health Sciences Center, Lubbock, TX, United States

**Keywords:** functional food, central nervous system, pain assessment, leaky gut, animals

## Abstract

**Objectives:** Emerging evidence suggests an important role of the gut-brain axis in the development of neuropathic pain (NP). We investigated the effects of gingerol-enriched ginger (GEG) on pain behaviors, as well as mRNA expressions of inflammation *via* tight junction proteins in GI tissues (colon) and brain tissues (amygdala, both left and right) in animals with NP.

**Methods:** Seventeen male rats were randomly divided into three groups: Sham, spinal nerve ligation (SNL, pain model), and SNL+0.375% GEG (wt/wt in diet) for 4 weeks. Mechanosensitivity was assessed by von Frey filament tests and hindpaw compression tests. Emotional responsiveness was measured from evoked audible and ultrasonic vocalizations. Ongoing spontaneous pain was measured in rodent grimace tests. Intestinal permeability was assessed by the lactulose/D-mannitol ratio in urine. The mRNA expression levels of neuroinflammation (NF-κB, TNF-α) in the colon and amygdala (right and left) were determined by qRT-PCR. Data was analyzed statistically.

**Results:** Compared to the sham group, the SNL group had significantly greater mechanosensitivity (von Frey and compression tests), emotional responsiveness (audible and ultrasonic vocalizations to innocuous and noxious mechanical stimuli), and spontaneous pain (rodent grimace tests). GEG supplementation significantly reduced mechanosensitivity, emotional responses, and spontaneous pain measures in SNL rats. GEG supplementation also tended to decrease SNL-induced intestinal permeability markers. The SNL group had increased mRNA expression of NF-κB and TNF-α in the right amygdala and colon; GEG supplementation mitigated these changes in SNL-treated rats.

**Conclusion:** This study suggests GEG supplementation palliated a variety of pain spectrum behaviors in a preclinical NP animal model. GEG also decreased SNL-induced intestinal permeability and neuroinflammation, which may explain the behavioral effects of GEG.

## Introduction

Neuropathic pain (NP) resulting from a lesion or disease of the somatosensory nervous system is a common chronic pain (Cavalli et al., 2019). NP in the general population is estimated to have a prevalence between 3% and 17% (Cavalli et al., 2019). NP is characterized by abnormal hypersensitivity to stimuli (hyperalgesia) and nociceptive responses to non-noxious stimuli (allodynia) (Colloca et al., 2017). Currently, available treatment options for NP are limited (Cavalli et al., 2019) and opioid analgesics have severe side effects and can result in opioid use disorder (Finnerup et al., 2015; Cooper et al., 2017).

Ample evidence shows NP is linked to excessive reactive oxygen species and inadequate endogenous antioxidants after nerve injury, resulting in neuroinflammation (Dai et al., 2020; Teixeira-Santos et al., 2020). Mitigating the neuroinflammation offers potential therapeutic targets in NP management. Dietary bioactive compounds have gained attention for NP and NP-related neuroinflammation due to their anti-inflammatory and anti-oxidant properties (Shen et al., 2022a). Therefore, the development and assessment of bioactive compounds for NP management could provide a new, safe, and effective analgesic alternative that is much needed.

A “leaky gut” refers to a damaged gut lining, which can no longer optimally function as a barrier, leading to an increase in the permeability of the intestinal mucosa along with low-grade inflammation (Yang et al., 2019; Lin et al., 2020). The link between leaky gut and neuroinflammation in NP and NP-associated behaviors has received increased attention, as shown by increased research into the permeability of the intestines (leaky gut) and the blood-brain barrier, followed by enhanced entrance of microbiota-produced substances into the peripheral and central nervous system (CNS) (Camilleri, 2019; Yang et al., 2019; Lin et al., 2020). Since the “leaky gut” may be linked to neuroinflammation, neuronal sensitization, and hyperexcitability in the development of NP, targeting the leaky gut using bioactive compounds *via* functional food or bioactive compounds may represent a new therapeutic strategy to manage NP.

Ginger (*Zingiber officinale* Roscoe) consists of a complex combination of biologically active constituents (6-, 8-, and 10-gingerol and 6-, 8-, and 10-shogaol) that contribute to ginger’s anti-inflammatory properties (Tjendraputra et al., 2001). Ginger and its bioactive components have been shown to penetrate the blood-brain barrier via passive diffusion, providing the basis for positive effects of ginger in the CNS (Simon et al., 2020). Ginger’s anti-nociceptive capabilities in a number of NP animal studies have been reviewed recently (Shen et al., 2022a). In brief, ginger consumption, in the forms of ginger extract, ginger essential oil, gingerols, and shogaols, has beneficial effects on NP-related parameters including mechanical allodynia and hyperalgesia, thermal and cold hyperalgesia, and anxiety-associated behaviors. Our team proposed that ginger’s underlying mechanisms include the suppression of glial cell and neutrophil activation, inhibition of expression/production of pro-inflammatory cytokines/chemokines, and reduction of circulating cell-free mitochondrial DNA levels (Shen et al., 2022a). However, no study has investigated how GEG would impact leaky gut and neuroinflammation via the gut-brain connection in NP status. For that we focused on a particular brain area, the amygdala, because of its link to NP mechanisms (Neugebauer, 2020) in brain signaling (Cowan et al., 2018).

We previously reported that GEG supplementation into the diet decreased mechanical hypersensitivity assessed in the von Frey test and favored microbiome composition and fecal metabolites (Shen et al., 2022b). Such beneficial effects of GEG may be due to its anti-inflammatory property as shown in the suppression of circulating cell-free mitochondrial DNA (Shen et al., 2022b). The current study advances this concept by investigating 1) if GEG supplementation would affect different NP-related behaviors, including sensory (mechanical withdrawal thresholds), emotional-affective (audible and ultrasonic vocalization), and spontaneous components, 2) if GEG supplementation would improve leaky gut, and 3) if GEG supplementation would suppress neuroinflammation in the colon (gut) and amygdala (brain). We hypothesized that GEG supplementation into the diet would mitigate pain behaviors through alleviating leaky gut (decreasing intestinal instability) and suppressing neuroinflammation in the colon and amygdala of neuropathic rats (spinal nerve ligation model, SNL). We selected the gut (colon) and the brain (right amygdala and left amygdala) in order to explore the effects of ginger bioactive compounds on the gut-brain connection that is relevant to NP and provide the knowledge basis for the development of precision nutrition for NP management.

## Materials and Methods

### Animals and Treatments

Seventeen male Sprague-Dawley rats (4-5-week-old, 140–170 g, Envigo, Cumberland, VA, United States) were housed individually under a 12-h light-dark cycle with food and water *ad libitum*. All procedures were approved by the Institutional Animal Care and Use Committee at Texas Tech University Health Sciences Center.

We used the spinal nerve ligation (SNL) model to study neuropathic pain, which is widely used for the preclinical study of NP mechanisms and the development of new analgesic drugs/compounds. Lumbar spinal nerve (L5) ligation in this model results in acute hypersensitivity within 1 week that persists for multiple weeks (Chung et al., 2004). The SNL model is well-established in our laboratories (Ji et al., 2017; Ji et al., 2018; Ji and Neugebauer, 2019; Navratilova et al., 2019; Presto et al., 2021; Shen et al., 2022b). In brief, rats were anesthetized with isoflurane (2%–3%; precision vaporizer, Harvard Apparatus) and the left L5 spinal nerve was surgically exposed and tightly ligated using 6–0 sterile silk. In the sham-operated control group, the nerve was exposed but not ligated.

There were 3 treatment groups in this study: a Sham group, an SNL group, and an SNL + GEG at 0.375% in diet w/w (SNL + GEG) group. After 5 days of acclimatization, we randomly assigned rats to the Sham group (N = 6), SNL group (N = 6) and SNL + GEG group (N = 5). The Sham and SNL groups were given AIN-93G diet (catalog number #D10012G, Research Diet, Inc., New Brunswick, NJ, United States) for 4 weeks. The SNL + GEG group, after SNL induction, was given GEG at 0.375% (wt/wt diet) into AIN-93G diet for 4 weeks. At doses in the range of 100 mg/kg to 400 mg/kg GEG has been shown to reduce inflammation in rats in various inflammation models (Li et al., 2012; Mansour et al., 2019). Based on these studies, we selected a dose of 0.375% (weight/weight in diet) for our study in SNL-treated rats, which corresponds to ∼150 mg/kg for rats. Based on the results of gas chromatography-mass spectrometry, GEG consists of 18.7% 6-gingerol, 1.81% 8-gingerol, 2.86% 10-gingerol, 3.09% 6-shogoal, 0.39% 8-shogaol, and 0.41% 10-shogaol. GEG was obtained from Sabinsa, Inc., Piscataway, NJ. Body weight, food intake, and water consumption was recorded weekly.

### Assessment of Mechanosensitivity

Mechanical withdrawal thresholds of spinal nocifensive reflexes were measured on the left paw using Electronic von Frey Aesthesiometer (IITC Life Science, Woodland Hills, CA, United States) with a plastic tip in an exclusive testing area (catalog number 76-0488, Harvard Apparatus, Holliston, MA, United States) 1 day before-SNL and 1, 2, 3, and 4 weeks after respective treatments, as described in our previous studies (Ji et al., 2018; Shen et al., 2022b). The average of six measurements per subject, taken at least 30 s apart, was calculated.

### Assessment of Emotional Responses

The pain-related emotional responses were assessed as vocalizations at 1 day pre-surgery and 4 weeks after feeding intervention. Vocalizations in the audible (20 Hz-16 kHz) and ultrasonic (25 ± 4 kHz) ranges were measured as in our previous studies (Han et al., 2005; Kiritoshi et al., 2016; Mazzitelli and Neugebauer, 2019; Presto et al., 2021). Animals were briefly anesthetized with isoflurane and placed in a custom designed recording chamber (U.S. Patent 7,213,538). After habituation to the chamber for 10 min, vocalizations were evoked by brief (15 s) innocuous (500 g/30 mm2) and noxious (1,500 g/30 mm2) stimuli applied to the left hind paw, using a calibrated forcep with a force transducer displaying output in grams. A microphone connected to a preamplifier and a bat detector were used to measure audible and ultrasonic vocalizations, respectively. Signals were digitized with UltraVox interface (Noldus Information Technology, Leesburg, VA, United States). Vocalizations were recorded for 1 min starting with the onset of mechanical stimulation and analyzed using Ultravox 2.0 software (Noldus Information Technology). Vocalizations were measured twice in the same animal with a 10 min interval, and then averaged.

### Assessment of Spontaneous Pain

Grimace test scoring was performed before surgery (baseline) and after 4 weeks of feeding treatment intervention based on the published work (Sperry et al., 2018). The rats were placed in individual plexiglass chambers with home cage bedding in a quiet environment. Two video cameras (Sony Handycam HDR-CX455 9.2 megapixels with lenses Zeiss Vario-Tessar, Sony Corporation of America, New York, NY, United States) were placed on the outside perpendicular to the front and back of the chambers. Rats were video-recorded for a 10-min duration. For image extraction, still images were selected and retrieved every 2 s (1/2fps) out of a recorded video using customized Python scripts. Ten representative images (at least 30 s apart) of the rat’s face/body were then selected for scoring from all the images generated and were assigned a random number code. Grimace scoring was performed by 5 treatment-blinded experienced evaluators as described before (Sotocinal et al., 2011; Sperry et al., 2018). Each image was scored based on four action units (parameters): orbital tightening, nose bulge, ear position, and whisker change. A score from 0–2 (0 = not present, 1 = moderate, 2 = severe) was assigned to each facial unit. A mean of each action unit for all 10 images scored by 5 evaluators was obtained. We analyzed the scores for each parameter individually averaging across the evaluators and also average score for all parameters.

### Assessment of Intestinal Permeability

Intestinal permeability was evaluated by analyzing urinary lactulose and mannitol levels (Nguyen et al., 2019). After 4 weeks of intervention, a 2-ml fresh solution containing 60 mg/ml lactulose and 40 mg/ml D-mannitol were given to each rat by oral gavage. Rats were placed individually in metabolic cages and the rats had free access to food and water. Urine was collected over 24 h. Thymol (10% dissolved in isopropanol) was added to the collecting tubes to prevent degradation of urinary sugars due to bacterial growth. Collected urine samples were stored at −80C before assay. Concentrations of the lactulose and mannitol were measured using the EnzyChromTM Intestinal Permeability Assay Kit (EIPM-100, BioAssay System, Hayward, CA, United States) following manufacture’s instruction. Lactulose and D-Mannitol concentrations were calculated by subtracting the optical density (O.D) from each sample from its own blank (O.D.), dividing by the slope of the standard curve of each compound and multiplying by the dilution factor of samples. Data is presented by ratio lactulose/D-Mannitol.

### Sample Collection

At the end of study, the animals were anesthetized, euthanized, and their blood was drawn for plasma and serum collection. In addition, the colon and both right and left amygdala were collected, reserved in RNAlater, and stored at −80°C for later mRNA expression analyses.

### RNA Isolation and qRT-PCR

Total RNA was isolated from amygdala (right and left) and colon using the RNAzol RT (RN190, Molecular Research Center Inc., Cincinnati, Ohio, United States), BAN ratio 1:200 (BN191, Molecular Research Center, Cincinnati, Ohio, United States). Total RNA was quantified using nanodrop at 260 nm (Nanodrop one, Thermo Scientific) then reverse transcribed into cDNA using Maxima first strand cDNA synthesis kit synthesis with dsDNase (Thermo Scientific, K1672, Waltham, MA, United States) using the thermal cycler Bio-rad S1000 (Bio-Rad Laboratories, Inc., Hercules, CA, United States). qRT-PCR was performed on Quant Studio 12 K Flex real time PCR system (Life Technologies, 4470689, Carlsbad, CA, United States) using samples cDNA for amplification of target genes with β-actin as the control with Universal SYBR green supermix (Bio-rad Laboratories, Inc., 17251-24, Hercules, CA, United States).

The following genes were tested: inflammation markers (NFkβ, TNFα). The primer sequences used are: β-actin, forward: 5′-ACA ACC TTC TTG CAG CTC CTC C-3′; Reverse: 5′-TGA CCC ATA CCC ACC ATC ACA-3′. TNFα, forwards: 5′- GAA CTC CAG GCG GTG TCT GT-3′; reverse: 5′ - CTG AGT GTG AGG GTC TGG GC-3′. NF-kB, forward: 5′- CCT CCA CCC CGA CGT ATT GC-3′; reverse: 5′- GCC AAG GCC TGG TTT GAG AT-3′. All genes expressions were normalized to our control β-actin. Gene expression was calculated by the following formula: 2-(ΔCT*1,000) (Rao et al., 2013).

### Statistical Analysis

Results are presented as mean ± standard error of mean (SEM). For data of von Frey tests, mixed ANOVA followed by post-hoc Tukey test at each collection time was conducted to examine overall group difference (i.e., group effect), change over time (i.e., time effect), and group difference in this change (i.e., group-by-time interaction). For other data, one-way ANOVA was performed with post-hoc Tukey test. All analyses were conducted using SAS/STAT 9.4 (SAS Institute Inc.) and statistical significance was determined a 0.05 alpha level. Additionally, for some comparisons with 0.05 < *p* < 0.1, the symbol # is used to show a tendency.

## Results

### Gingerol-Enriched Ginger Supplementation Mitigated NP Hypersensitivity

**FIGURE 1:**
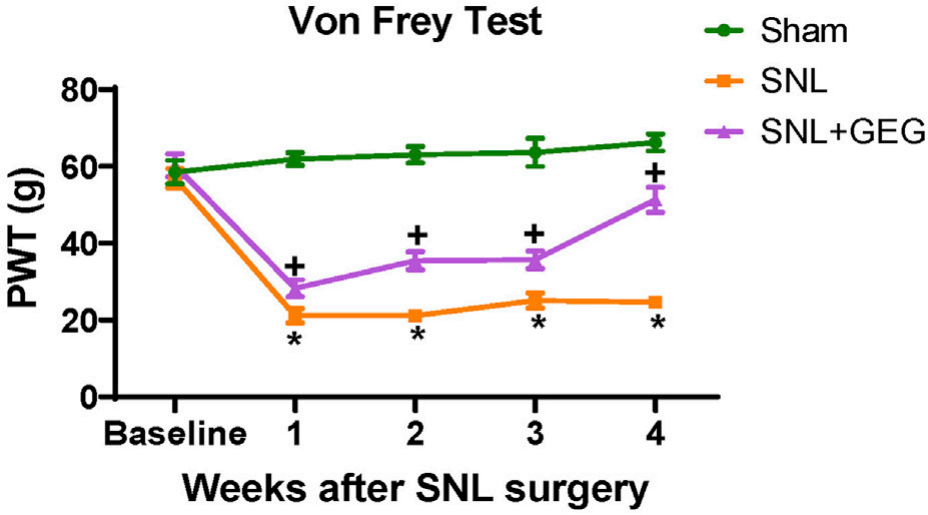
GEG increased the mechanical thresholds of SNL rats as assessed by an electronic von Frey anesthesiometer. Data is expressed as mean ± SEM. *n* = 5–6 per group. Data was analyzed by mixed ANOVA followed by a *post hoc* Tukey’s test. *p < 0.05 vs. Sham. ^+^ *p* < 0.05 vs. SNL.

Mechanical hypersensitivity was assessed in the von Frey test (Figure 1). The interaction effect was significant [F(3, 42) = 18.51, *p* < .0001], confirming that mechanosensitivity changed uniquely for different groups as shown in Figure 1. The post-hoc pairwise group comparisons were all significant at each time point (all adjusted *p* < .05)—the only exceptions were the difference between SNL and SNL + GEG at week 1 and week 3. Compared with the sham group, the SNL group had significantly greater mechanosensitivity starting at 1-week post-operation and persisting throughout the observation period (4 weeks after SNL induction). GEG supplementation significantly decreased hypersensitivity in the treatment group (SNL + GEG) relative to the SNL group as early as 1-week post-operation, and the effect persisted for 4 weeks, as shown by increased mechanical thresholds. At the end of the study (4 weeks after supplements started), the order of pain sensitivity was SNL group > SNL + GEG group > sham group.

**FIGURE 2:**
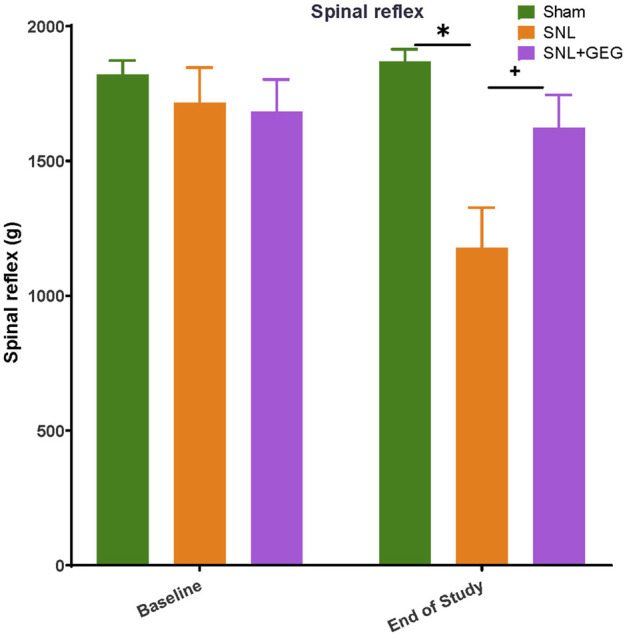
GEG increased the mechanical thresholds of SNL rats as assessed by compression of the hind paw with calibrated forceps. Data is expressed as mean ± SEM. *n* = 5–6 per group. Data was analyzed by one-way ANOVA followed by a *post hoc* Tukey’s test at each collection time. *p < 0.05 vs Sham. ^+^ *p* < 0.05 vs SNL.

Mechanical hypersensitivity was assessed in the left hind paw by a compression test (Figure 2). In the hind paw compression test, there were no significant differences in mechanical withdrawal thresholds (spinal reflex, g) among all groups at the baseline (Figure 2). 4 weeks postinduction of the spinal ligation procedure, the SNL group had significantly lower thresholds compared to the sham group. Supplementation of GEG into the diet increased mechanical thresholds in the SNL group significantly.

### Gingerol-Enriched Ginger Supplementation Decreased NP-Induced Emotional Responses

**FIGURE 3:**
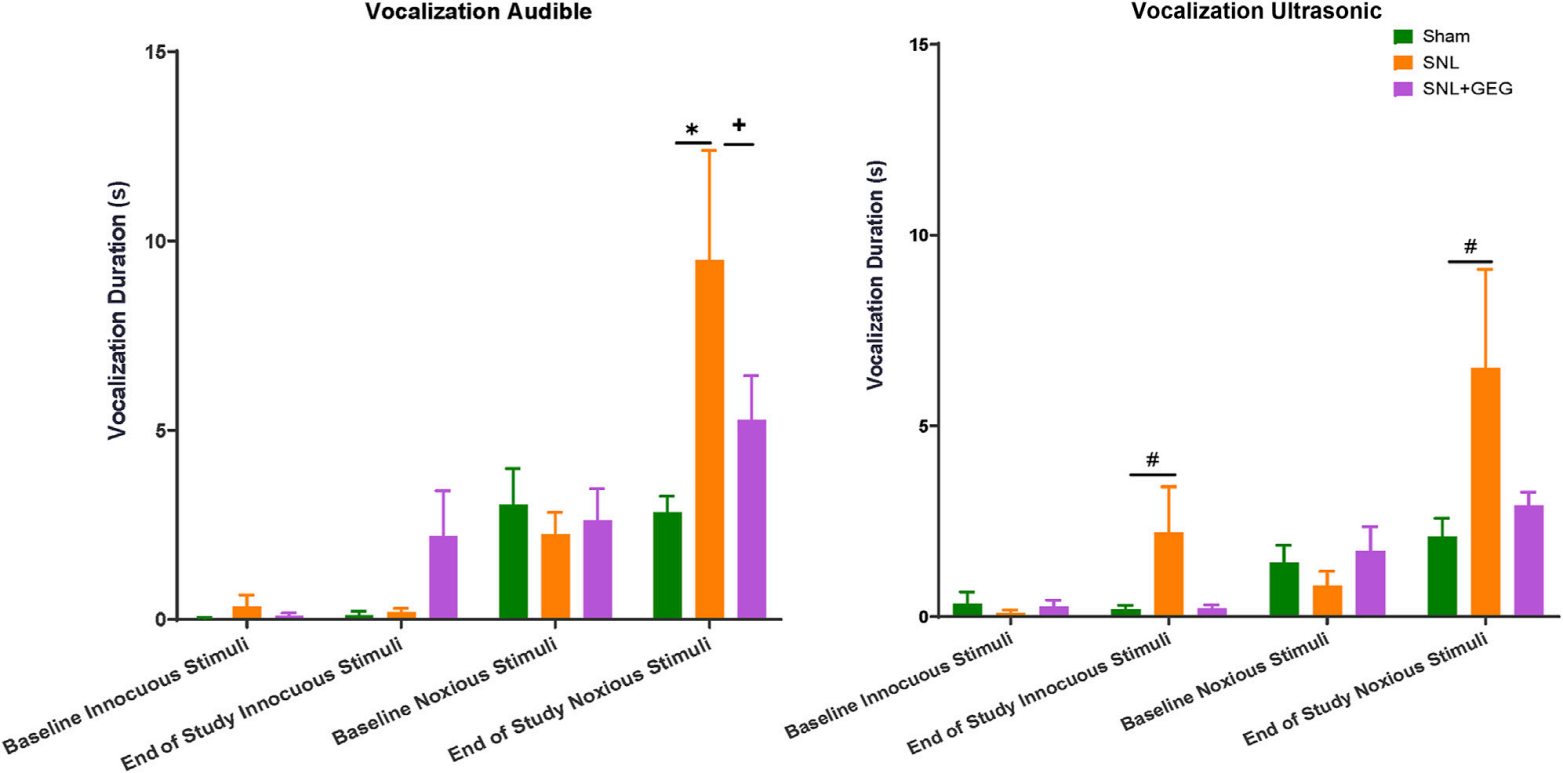
Effect of GEG supplementation on audible, pain-induced vocalizations in response to innocuous stimuli (left) and noxious stimuli (right). Data is expressed as mean ± SEM. *n* = 5–6 per group. Data was analyzed by one-way ANOVA followed by a *post hoc* Tukey’s test at each time point. *p < 0.05. vs Sham. ^#^0.05 < *p* < 0.1 vs Sham. ^+^ *p* < 0.05 vs SNL.

GEG supplementation tended to reduce audible and ultrasonic vocalizations in response to innocuous and noxious stimuli. Effects of GEG supplementation on pain-related emotional responses were measured as audible and ultrasonic vocalizations in response to innocuous and noxious mechanical stimuli (Figure 3). At baseline, there were no differences in the duration of audible and ultrasonic vocalizations evoked by innocuous and noxious stimuli among all treatment groups. At the end of the study (4 weeks post-induction of SNL), the SNL group showed increased audible and ultrasonic vocalizations to innocuous and noxious mechanical stimuli compared to the sham group. Supplementation of GEG to the diet reduced the duration of audible vocalizations in SNL rats significantly, while the other outcome measures showed a trend of inhibition by GEG.

### Gingerol-Enriched Ginger Supplementation Decreased NP-Induced Spontaneous Pain Behaviors

**FIGURE 4:**
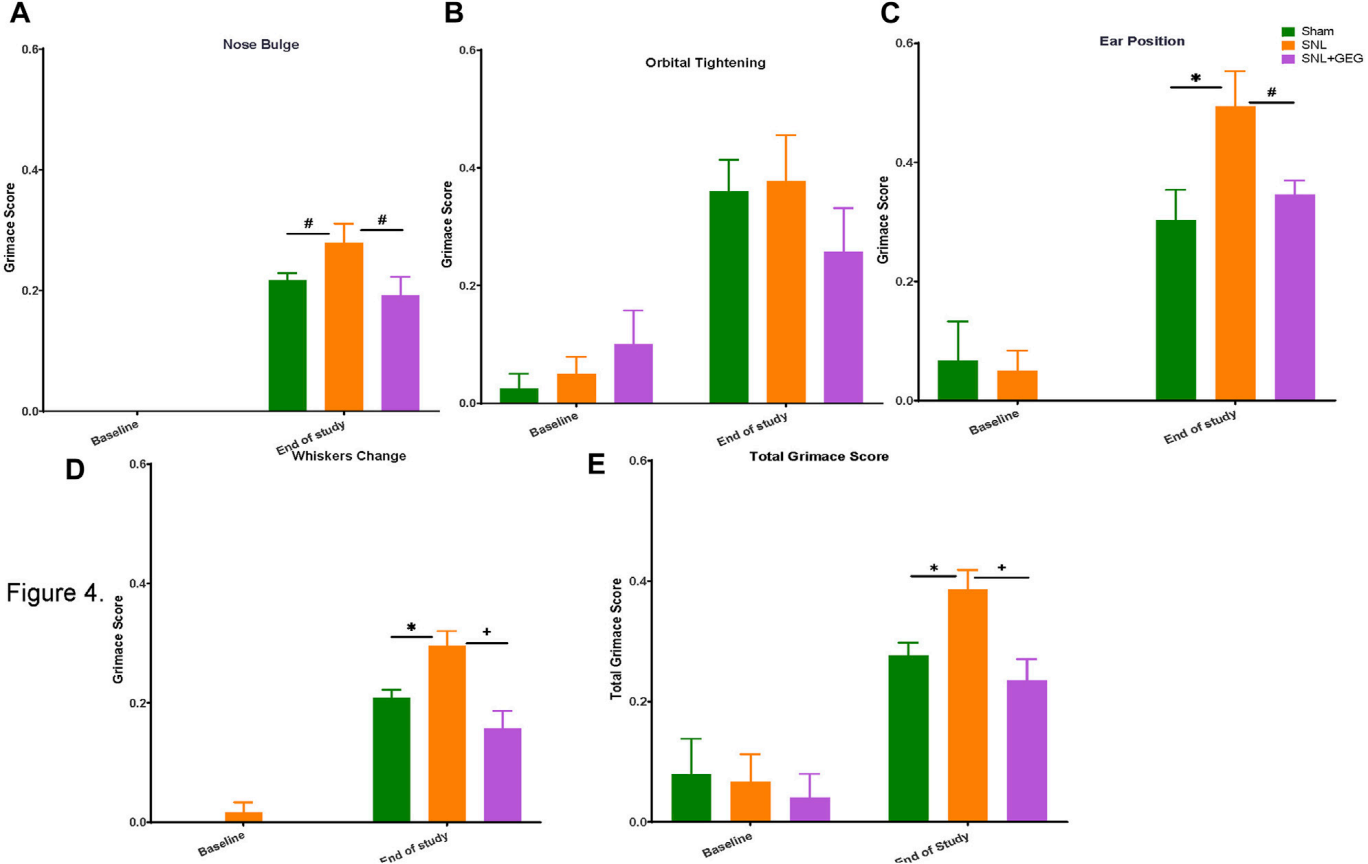
Effect of GEG supplementation on spontaneous pain in SNL rats as assessed by the rodent grimace test, showing scores for nose bulge **(A)**, orbital tightening **(B)**, ear position **(C)**, and whisker change **(D)**; total **(E)**. Data is expressed as mean ± SEM. *n* = 5–6 per group. Data was analyzed by one-way ANOVA followed by a *post hoc* Tukey’s test at each collection time. *p < 0.05 vs Sham. ^#^0.05 < *p* < 0.1 vs Sham or vs SNL. ^+^ *p* < 0.05 vs SNL.

GEG supplementation reduced SNL-induced spontaneous pain in nose bulge, ears position, whisker change, and total score of rats. Non-evoked ongoing/spontaneous pain was assessed in the rodent grimace test for nose bulge (Figure 4A), orbital tightening (Figure 4B), ear position (Figure 4C), whisker change (Figure 4D), and total score (Figure 4E). At baseline, there were no significant differences between the 3 experimental groups in any parameters of spontaneous pain measured (nose bulge, orbital tightening, ear position, whisker change, and total score; *p* > 0.05). Some scores were rated “0” by coders at the baseline. 4 weeks after SNL induction, spontaneous pain was detected in nose bulge, ear position, whisker change, and total score, while there was no significant change in orbital tightening (*p* > 0.05). GEG supplementation for 4 weeks significantly mitigated SNL-induced spontaneous pain evidenced in significantly nose bulge, ear position, whiskers change, and total score of SNL-operated rats.

### Gingerol-Enriched Ginger Supplementation Tended to Improve Intestinal Integrity in NP

**FIGURE 5:**
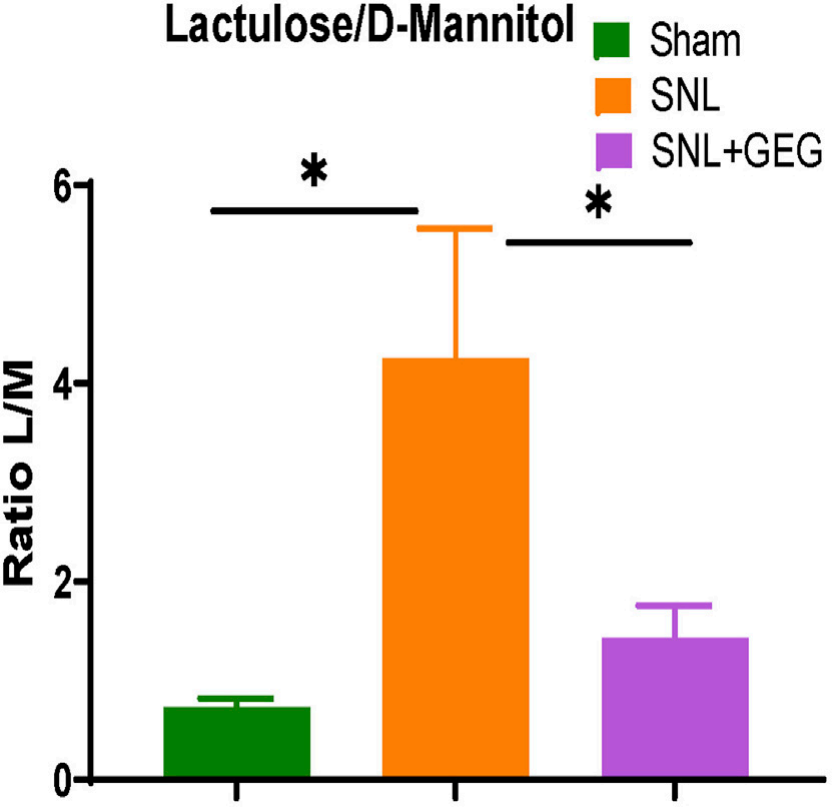
Effect of GEG supplementation on intestinal permeability in SNL rats. The ratio of lactulose to D-mannitol (L/M) increased in the NP state. Data is expressed as mean ± SEM. *n* = 4 per group. Data was analyzed by one-way ANOVA followed by a *post hoc* Tukey’s test. *p < 0.05.

Four weeks after SNL induction, there was an increase in intestinal permeability as shown in increased ratio of lactulose/D-mannitol concentrations in the urine of animals (Figure 5). GEG supplementation significantly decreased the lactulose/D-mannitol ratio, suggesting decreased intestinal permeability.

### Gingerol-Enriched Ginger Supplementation Decreased mRNA Expression in Amygdala and Colon in NP

**FIGURE 6:**
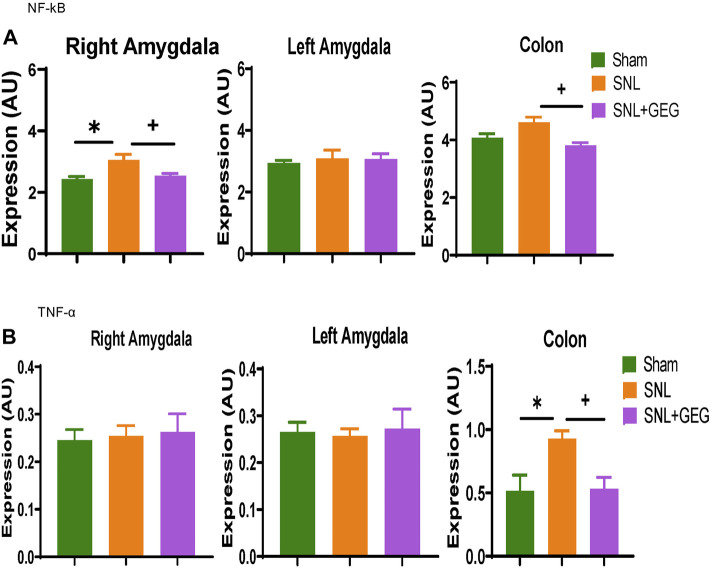
Effect of GEG supplementation on mRNA expression of NF-κB **(A)** and TNF-α **(B)** in the right amygdala, left amygdala, and colon of SNL rats. Data is expressed as mean ± SEM. n = 5-6 per group. Data was analyzed by one-way ANOVA followed by a *post hoc* Tukey’s test. *p < 0.05 vs Sham. ^+^ *p* < 0.05 vs SNL.

Effects of GEG supplementation on the mRNA expression of neuroinflammation markers NF-κB (Figure 6A) and TNF-α (Figure 6B) were assessed in the colon as well as right and left amygdala in the NP condition. Compared to the Sham group, the SNL group showed significantly increased mRNA expression of NF-κB in the right amygdala and colon. Supplementation of GEG into diet significantly decreased the NF-κB mRNA expression levels in the right amygdala and colon of SNL rats. Similar to the effects on NF-κB mRNA, TNF-α mRNA expression found in the colon of SNL rats also significantly increased, while GEG supplementation significantly decreased the SNL-induced TNF-α mRNA expression changes in colon.

### Disscusion

This study shows for the first time that dietary GEG supplementation mitigated NP-induced sensory and affective pain-related behaviors in animals with NP, and these effects may involve GEG’s impacts on intestinal permeability and neuroinflammation in the colon (gut) and amygdala (brain). The results provide evidence for beneficial behavioral effects of GEG through the modulation of the brain-gut axis.

Natural or extracted ginger bioactive compounds (i.e., *Z. officinale* Roscoe rhizome extract, zerumbone, red ginger oil, shogaol-enriched ginger root extract, and 6-GEG), have been shown to mitigate mechanical allodynia and thermal hyperalgesia in animals with diabetic NP or surgical NP (Shen et al., 2022b). To the best of our knowledge, the present study is the first study to assess dietary GEG on a variety of pain-related behaviors using different pain assessment methods. The results for the sensory, emotional and spontaneous aspects of NP not only corroborate our previous study on sensory pain using the von Frey test (Shen et al., 2022b), but also expand our understanding of the breadth of the pain behavioral effects of GEG on NP.

Pain behavioral studies are a fundamental tool for the validation of pain mechanisms and for the assessment of potential nutraceutical approaches in NP management. In addition to sensory pain, we also examined emotional aspects of pain measuring vocalizations. The analysis of audible and ultrasonic vocalizations in response to noxious stimuli is a well-established approach to assess supraspinally organized pain behaviors (Portfors, 2007). Vocalizations in the audible range represent a nocifensive response, whereas ultrasonic vocalizations in the 25 kHz range reflect negative emotional-affective behavior (Han et al., 2005; Neugebauer et al., 2007; Presto et al., 2021). Therefore, vocalizations provide additional important information about higher integrated pain behaviors. The computerized analysis of audible and ultrasonic vocalization is a valid, quantitative, reliable, and convenient method, having been successfully employed in various pain models, including NP (Ji et al., 2017; Ji et al., 2018; Presto et al., 2021; Mazzitelli et al., 2022). In the present study, we also found increased audible and ultrasonic vocalization of SNL rats to innocuous and noxious stimuli compared to sham rats, which may reflect allodynic and hyperalgesic components of NP. Importantly, this study demonstrates that GEG supplementation decreased vocalizations. Though these effects did not reach the level of statistical significance, they further support an anti-allodynic effect of GEG in NP. The large variability (reflected in the standard error of mean) and small sample size could explain the lack of statistically significant differences between the SNL group and the SNL + GEG group. Further studies including more animals for vocalization assessment are needed to confirm the effects of GEG.

Another important aspect of pain is non-evoked spontaneous or ongoing pain. Evoked pain occurs in response to peripheral stimuli and can be categorized as hyperalgesia, in which sensitivity to noxious stimuli is increased, or allodynia, in which innocuous stimuli become noxious. Spontaneous NP occurs independently of external stimuli and is described by patients as an intermittent, burning or stabbing sensation commonly rated as severe (Schneider et al., 2017). In this study, we evaluated not only evoked pain (mechanical hypersensitivity and emotional responses) but also spontaneous pain using the rodent grimace scale test. In addition to mitigating evoked pain (see above), GEG also decreased various grimace metrics, suggesting beneficial/mitigating effects of GEG on clinically relevant spontaneous NP.

Neurobiological mechanisms include emotional network plasticity in the corticolimbic system (Meier et al., 2017; Elman and Borsook, 2018; Nees and Becker, 2018). Specifically, the amygdala, a limbic structure, has emerged as a key player in the emotional-affective dimensions of pain and pain modulation (Veinante et al., 2013; Thompson and Neugebauer, 2019; Neugebauer, 2020). It is commonly believed that the interaction in the gut-brain axis is bi-directional (Lin et al., 2020). Our observations that SNL increased intestinal permeability and neuroinflammatory markers in the colon (gut) and amygdala (brain) support the critical role of the amygdala in brain-gut signaling as a neurobiological mechanism of NP.

There is evidence for right-hemispheric lateralization of amygdala function in pain, with the right amygdala being pain facilitatory and the left amygdala serving “anti-nociceptive” functions (Allen et al., 2021). Our study is the first study to demonstrate the beneficial effects of GEG in NP development *via* the gut-brain connection through actions in the amygdala and the improvement of leaky gut syndrome.

Neuroinflammation comprises activation of glial cells and mitochondrial dysfunction in the peripheral and central nervous system, leading to the release of proinflammatory cytokines and chemokines that have been implicated in pain mechanisms (Donnelly et al., 2020; Tan et al., 2021). Thus, the suppression of neuroinflammation by targeting proinflammatory cytokine and chemokine signaling would be a promising strategy to alleviate or prevent NP states. In the development of NP, NF-κB has been shown to trigger a self-perpetuating process resulting in progressive NP (Liu et al., 2017). Enhanced production of the cytokine TNF-α in the brain (locus coeruleus and hippocampus) also occurs during NP development (Sud et al., 2008). Intriguingly, the inhibition of SNL-induced increase in NF-κB and TNF-α mRNA expression in the right amygdala and colon by GEG supplementation found in this study, may, therefore, represent a mechanism of GEG’s beneficial effect for the treatment of NP, again through the gut-brain axis.

There are study limitations in the present study. In order to confirm the connection between leaky gut (intestinal permeability), blood-brain barrier integrity, and tight junction proteins expression, sufficiently powered studies are warranted to assess not only mRNA but also protein expression of tight junction proteins in brain and GI tissues. This study focused on how GEG affects the changes in mRNA expression levels that are related to neuroinflammation in the amygdala and colon. We did not perform any histopathological assessment in the colon or amygdala to evaluate possible changes in glial cells with GEG. To the best of our knowledge, the effect of GEG on the number or morphology of glial cells in either colon or amygdala has not yet been evaluated. This important knowledge gaps remains to be addressed in future studies.

## Conclusion

GEG supplementation into the diet decreased NP-related pain behaviors, namely mechanosensitivity, emotional pain responses, and spontaneous pain. GEG supplementation also decreased intestinal permeability as well as the mRNA expression of neuroinflammatory factors in both amygdala and colon, suggesting GEG has a beneficial impact on NP development via the gut-brain axis.

## Data Availability

The original contributions presented in the study are included in the article/supplementary material, further inquiries can be directed to the corresponding author.
